# Enhanced Peelability and Quality of Whiteleg Shrimp (*Litopenaeus vannamei*) Using Pulsed Electric Field (PEF) Treatment

**DOI:** 10.3390/foods14020148

**Published:** 2025-01-07

**Authors:** Gyeong-Seo Park, Hyeon Seo, Han-Baek Lee, Ji-Won Lee, Hafiz Muhammad Shahbaz, Se-Ho Jeong, Dong-Un Lee

**Affiliations:** 1 Department of Food Science and Technology, Chung-Ang University, Anseong 17546, Republic of Korea; yuni2070@naver.com (G.-S.P.); clear0330@naver.com (H.S.); hanbaek98@naver.com (H.-B.L.); melona7237@naver.com (J.-W.L.); calvin0223@naver.com (S.-H.J.); 2 Department of Nutrition and Health, College of Medicine and Health Sciences, United Arab Emirates University, Al Ain 15551, United Arab Emirates; shahbaz@uaeu.ac.ae

**Keywords:** pulsed electric field, shrimp peeling, texture analysis, electroporation, non-thermal processing

## Abstract

This study investigated the effects of pulsed electric field (PEF) treatment on the peeling efficiency and textural properties of whiteleg shrimp (*Litopenaeus vannamei*). Shrimp samples were treated at field strengths of 0, 1.0, 1.5, and 2.0 kV/cm to assess PEF impact on peeling force, incomplete peeling percentage, and texture profile. The results showed that PEF treatment significantly reduced the peeling force from 50.88 N in controls to 42.99 N at 2.0 kV/cm, while the percentage of incompletely peeled shrimp decreased from 27.5% to 15.9%. Texture profile analysis indicated that PEF treatment had no impact on the key properties of hardness and chewiness (no significant difference), with a reduction in springiness observed at higher field strengths. Improvements in peelability are attributed to electroporation, which disrupts collagen in the connective tissue between the shrimp shell and muscle. These findings indicate that PEF treatment is an efficient, non-thermal method for enhancing shrimp peeling processes while preserving textural integrity. PEF technology offers a promising alternative to traditional mechanical and thermal methods in the seafood processing industry.

## 1. Introduction

The whiteleg shrimp (*Litopenaeus vannamei*) is one of the most widely consumed seafoods globally, valued for its high content of amino acids, polyunsaturated fatty acids, and other essential nutrients [1]. The Global Shrimp Aquaculture Production Survey projected a 4.8 percent growth in world farmed shrimp production in 2024, reaching approximately 5.88 million metric tons [2]. Growing consumer demand for convenient and ready-to-eat seafood products has driven rapid expansion in the shrimp processing industry, which now represents approximately 45% of the global seafood processing market [3]. A significant portion of this market is linked with peeled shrimp, a product that requires efficient and effective processing methods [4].

Shrimp peeling presents several challenges due to the tight attachment of the shell to the muscle by connective fibers, making shell removal a labor-intensive process. Traditionally, shrimp are peeled either manually or mechanically, both of which can lead to substantial shrimp meat loss and high labor costs [5]. To improve efficiency and reduce waste, the seafood industry has explored the innovative techniques of high-pressure processing (HPP), ultrasound, and enzymatic treatments [6,7], with each method aiming to loosen the shell while preserving the texture and quality of the shrimp meat.

HPP has been effective in loosening shrimp shells through changes in the structure of collagen in the shrimp epidermis. This leads to easier peeling but may affect the textural properties of the meat [6,8]. Ultrasound technology uses high-frequency sound waves to induce cavitation to loosen the shrimp shell by creating small pores and facilitating mass transfer between the muscle and shell; however, while effective, ultrasound may also impact the integrity of the shrimp meat if not properly controlled [7]. Enzymatic treatments break down proteins in the attachment fibers, allowing the shell to be removed with minimal mechanical force [9]; however, the widespread use of enzymes in the seafood industry is limited by cost and a potential impact on the sensory qualities of shrimp.

In recent years, pulsed electric field (PEF) technology has emerged as a promising non-thermal method for food processing [10,11,12]. The PEF method applies short bursts of a high-intensity electric field to food products, causing electroporation, which creates pores in cell membranes and facilitates mass transfer without significantly affecting the enzymatic or structural integrity of the food [13]. PEF technology has been successfully applied to fruits and vegetables, where it aids in drying, extraction, and peeling [11]. For example, PEF technology has been used to enhance the peeling of tomatoes and peaches while reducing mechanical damage and improving processing efficiency [10]. However, its application in seafood, particularly in shrimp processing, remains relatively unexplored. Most studies have focused on plant-based foods, and no significant investigations have been conducted on the application of the PEF method in shrimp peeling [14]. The present study aims to fill this gap by exploring the effects of the PEF method on the peeling and quality characteristics of whiteleg shrimp.

## 2. Materials and Methods

### 2.1. Shrimp Preparation

Live whiteleg shrimp (*Litopenaeus vannamei*) measuring 16–18 cm in length and weighing 40–60 shrimp per kg were sourced from an indoor aquaculture facility, Freshrim (Ansan-Si, Republic of Korea). Upon harvesting, shrimp were immediately packed in ice and transported to the laboratory within 1 h to ensure freshness. Upon arrival, shrimp were thoroughly washed with cold water to remove impurities, and excess water was drained. Shrimp were prepared for experiment within 1 h of arrival by removing legs and excising two segments from the first abdominal section. This preparation step was performed to ensure uniformity across all samples used for PEF treatment and subsequent analysis.

### 2.2. PEF Treatment

PEF treatment was administered using a 5 kW pulse generator (HVP-5, DIL, Quakenbruck, Germany) equipped with a batch chamber comprising two parallel electrodes separated by an 80 mm gap. Four shrimp abdominal sections were placed in the chamber with 200 mL of tap water as the processing medium. Shrimp samples were subjected to PEF treatment under the following electric field strengths: 0 kV/cm (control); 1.0 kV/cm (PEF_1.0); 1.5 kV/cm (PEF_1.5); and 2.0 kV/cm (PEF_2.0). The pulse width was maintained at 20 μs with a pulse frequency of 100 Hz, and a total of 500 pulses were applied for each treatment. A total of 20 shrimp were utilized for each treatment condition, resulting in 40 abdominal sections (two sections per shrimp). This ensured sufficient replicates for statistical analysis while maintaining consistency across experimental groups.

### 2.3. Measurement of Peelability After PEF Treatment

The peelability of treated shrimp was evaluated using a texture analyzer (TAHDi/500, TAHD, London, UK) following the methodology described by Dang et al. [7]. A total of 40 shrimp samples were used for this analysis. Briefly, each sample of two abdominal segments was weighed, affixed to a pin in the probe, and clipped into the texture analyzer to perform tension tests (Video S1). These tests were designed to quantify the force required to peel the shrimp shell from the meat. Peeling work was defined as the product of the force applied (N) and the distance traveled (mm) to remove the shell divided by the weight of the two segments (g) using Equation (1). The percentage of incompletely peeled shrimp was calculated as the ratio of the total number of shrimps with shells remaining attached to the total number of shrimps tested, as shown in Equation (2).

(1) $$W=\frac{Fd}{m_{i}}$$

where *W* (mJ/g) is the peeling work, *F* (N) is the force required to peel the shrimp shell, *d* (mm) is the distance required to remove the shell, and mi is the weight of two shrimp segments.

(2) $$Incompletely\;peeled\;(\%)=\frac{Number\;of\;shrimp\;having\;any\;shell\;attached}{Total\;number\;of\;shrimp\;used\;for\;TA\;peeling}\times 100$$

### 2.4. Measurement of Texture Properties of Treated Shrimp

We performed a texture profile analysis (TPA) to determine the texture properties of the shrimp samples, using a texture analyzer (TAHDi/500, TAHD, UK) as outlined by Lin et al. [15]. A total of 40 abdominal sections per treatment group, as described in Section 2.2, were prepared for TPA and cutting force tests. For these tests, 14 sections with nearly identical sizes were selected to ensure consistency in the cross-sectional area and minimize variability. Following the peeling process, shrimp abdominal sections were subjected to TPA using a P/75 probe under controlled conditions with a pre-, test, and post-test speed of 1.0 mm/s, a compression ratio of 50%, and a trigger force of 0.02 N. TPA measured key texture attributes, including hardness, springiness, and cohesiveness. In addition to TPA, cutting force was measured using a Warner–Bratzler flat blade attached to the texture analyzer. The cutting force required to cut through shrimp samples horizontally was recorded, based on the methodology used by Boonsumrej et al. [16] with minor modification. The maximum force (N) was measured on 14 shrimp abdominal sections with a pre-test speed of 5.0 mm/s, a test and post-test speed of 2.0 mm/s, and a trigger force of 4.9 N.

### 2.5. Statistical Analysis

All experimental data were analyzed using a one-way analysis of variance (ANOVA). A significance level of *p* < 0.05 was applied. Post hoc comparisons were performed using Duncan’s multiple range test to identify significant differences between treatment groups. Statistical analyses were conducted using IBM Statistical Package for the Social Science (SPSS) software version 20 (IBM Corp., Armonk, NY, USA). Results were reported as means ± standard deviations.

## 3. Results and Discussion

### 3.1. Effect of PEF on Shrimp Peelability

**Figure 1:**
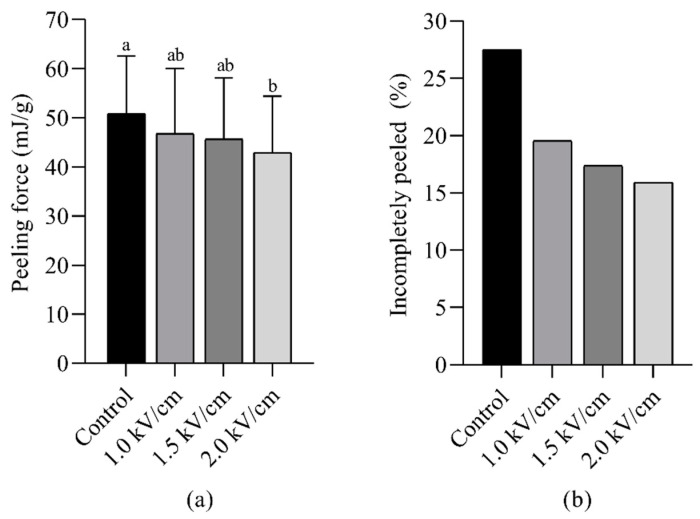
Peeling force (**a**) and incompletely peeled percentage (**b**) for shrimp after PEF treatment with different field strengths (n = 40). The error bar for the peeling force values (**a**) represents standard deviation, and letters (**a**,**b**) indicate significant differences for each sample (*p* < 0.05).

**Figure 2:**
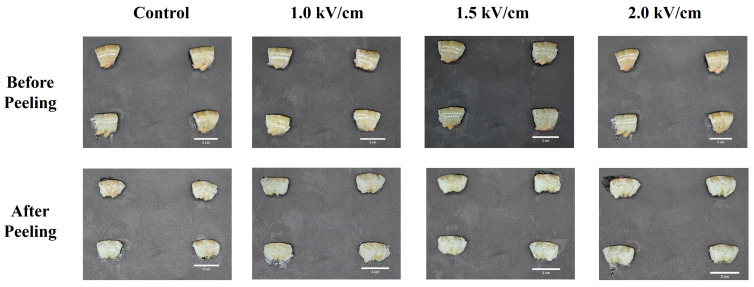
Appearance of PEF-treated shrimp before and after peeling. The scale bar of the images is 3 cm.

The effect of PEF treatment on the peelability of shrimp is shown in Figure 1. As the intensity of the PEF treatment increased, both the peeling force and the percentage of incompletely peeled shrimp decreased. The peeling force was reduced from 50.88 ± 11.7 N in the control group to 42.99 ± 11.4 N at a PEF strength of 2.0 kV/cm. Similarly, the percentage of incompletely peeled shrimp decreased from 27.5% in the control group to 15.9% for the PEF 2.0 treatment. Furthermore, PEF treatment had no discernible effect on shrimp appearance before or after peeling (Figure 2).

The improvement in peeling efficiency observed in this study is consistent with earlier findings that non-thermal HPP and ultrasound treatments can modify the structural integrity of the connective tissue between the shrimp shell and muscle [4,5]. Collagen, a major component of this connective tissue, is susceptible to degradation under physical treatments. In the case of PEF treatment, electroporation likely disrupts collagen fibers and facilitates easier shell detachment, as reported by Gómez et al. (2019) for other food matrices undergoing PEF treatment. This mechanism parallels that of ultrasound-induced vapor cavitation, which generates bubbles within the connective tissue to increase peeling efficiency [7].

Notably, results from this study support findings from HPP treatment studies on shrimp, whereby collagen denaturation at higher pressures significantly enhanced peelability [4]. Similarly, previous work by Xu et al. [17] on ice water pretreatment of shrimp found that the mechanical disruption of muscle fibers under controlled conditions improved shell removal without compromising product quality. These findings suggest that PEF treatment has a comparable effect by causing minor disruptions in shrimp epidermal collagen, allowing for easier separation of the shell from the muscle.

### 3.2. Textural Properties of PEF-Treated Shrimp

Table 1 summarizes the texture profile analysis (TPA) and cutting force results for shrimp after PEF treatment. No significant differences were found between the control and PEF-treated samples for hardness, cohesiveness, adhesiveness, gumminess, chewiness, resilience, or cutting force. However, a notable decrease in springiness was observed as the intensity of PEF treatment increased. Springiness values decreased by 9.9% at PEF 1.0, 8.7% at PEF 1.5, and 17.9% at PEF 2.0 compared to controls.

The reduction in springiness observed in this study may be linked to structural changes in shrimp muscle fibers caused by electro-permeabilization during PEF treatment. Similar effects were reported by Gómez et al. [18], who observed that PEF treatment can loosen muscle fibers and reduce springiness in meat tissues. The extent of electro-permeabilization depends on the strength of the electric field applied, with higher intensities causing greater disruption to muscle fibers. This could explain the progressive decrease in springiness with increasing PEF intensity in this study. However, studies on the PEF treatment of abalone and turkey breast meat by Arroyo et al. [19] and Luo et al. [20] showed that the protein structure of muscle fibers was not significantly affected, suggesting that the effect of PEF treatment on textural properties may vary depending on the particular food matrix. While springiness was affected by PEF treatment, the lack of significant changes in the textural parameters of hardness and chewiness is noteworthy for maintaining the sensory quality of shrimp. Textural properties such as hardness and chewiness are critical factors in consumer acceptance [17]. Additionally, the cutting force results indicate that PEF-treated shrimp retained structural integrity, which is beneficial for processing and handling.

The observed phenomena can be attributed to disparities in the chemical composition of the muscle and shell. The moisture and protein content of shrimp are higher in the muscle than in the shell, resulting in higher electrical conductivity [21,22]. The lower electrical conductivity of the shell and its connecting fibers may lead to localized intensification of the electric field, enhancing the electroporation effect in these regions. This phenomenon could explain the selective disruption of connective tissue near the shell while the structural integrity of the deeper muscle layers is preserved. The PEF effect is contingent on the electrical conductivity of tissues, and lower conductivity increases the electric field strength under the same energy. In this study, the peel part was positioned horizontally with the electrode, enabling the pulse energy to act on the peel and subsequently transfer to the deeper muscle layers. Consequently, there might be a discrepancy in the PEF effect between the peel and muscle, which could explain the observed ease of peeling. However, the effect’s limitations in properties like hardness and cutting force should be noted.

The retention of textural properties such as hardness and chewiness alongside the improved peeling efficiency ensures that PEF-treated shrimp meet both consumer expectations for quality and industry requirements for processing efficiency. These findings highlight the efficacy of PEF treatment in enhancing processing efficiency while preserving critical quality attributes, positioning it as a promising approach for use in the seafood industry. Future studies should systematically investigate the role of electrical conductivity and tissue composition in influencing the selective effects of PEF treatment, particularly to optimize its application across different seafood matrices.

### 3.3. Mechanism and Industrial Implications

This study demonstrates that PEF treatment enhances shrimp peeling efficiency while maintaining key textural properties, offering a promising, non-thermal alternative to labor-intensive conventional methods [5]. Its potential for minimizing meat loss and serving as a complementary tool to existing peeling methods underscores its value for the shrimp processing industry by providing energy-efficient and cost-effective solutions.

In comparison with enzymatic and ultrasonic methods, PEF treatment offers the advantage of being non-thermal, thus preserving the freshness and quality of shrimp. As reported by Koch et al. [11], non-thermal processing technologies are gaining traction in the food industry due to their ability to enhance food quality while reducing energy consumption. For example, PEF treatment has been shown to improve peeling efficiency in tomatoes and peaches [10]. Its application in seafood offers potential for improving processing efficiency in this sector.

Additionally, the potential of PEF treatment to preserve the textural properties of shrimp could address consumer demand for high-quality, minimally processed seafood products. This is particularly relevant in the context of the growing market for convenience seafood, where ready-to-eat and easy-to-peel shrimp are highly desired [4]. Furthermore, the non-invasive nature of PEF treatment could complement existing processing technologies, such as HPP or enzymatic treatments, offering a more versatile approach to seafood processing.

Although the current study demonstrates the benefits of PEF treatment in shrimp processing, further research is needed to optimize PEF conditions for different shrimp sizes and species. Future studies could investigate synergies between PEF and other non-thermal technologies, such as ultrasound or cold plasma, to further enhance processing efficiency. Moreover, investigations into the long-term effects of PEF treatment on shrimp quality during storage would provide valuable insights for applications in industrial settings.

## 4. Conclusions

PEF treatment significantly improved the peeling efficiency of whiteleg shrimp by reducing the percentage of incompletely peeled shrimp and the peeling force required. Additionally, PEF treatment maintained key textural properties and ensured shrimp quality. These findings indicate that PEF is an effective, non-thermal method for enhancing shrimp processing efficiency while maintaining product integrity; however, further investigations are required to better understand the impact of PEF treatment on shrimp connective tissue and develop optimized protocols for various shrimp species.

## Figures and Tables

**Table 1:** Texture profile analysis of shrimp for different PEF strengths.

Field Strength (kV/cm)	Texture Properties							
	Hardness (N)	Adhesiveness	Springiness	Cohesiveness	Gumminess	Chewiness (N)	Resilience	Cutting Force (N)
Control	46.49 ± 8.19 ^a^	−33.88 ± 12.12 ^a^	0.62 ± 0.11 ^a^	0.57 ± 0.12 ^a^	26.81 ± 7.23 ^a^	17.39 ± 8.85 ^a^	0.37 ± 0.07 ^a^	19.54 ± 2.84 ^a^
PEF_1.0	47.37 ± 5.63 ^a^	−28.22 ± 6.42 ^a^	0.56 ± 0.04 ^ab^	0.50 ± 0.03 ^a^	23.81 ± 3.95 ^a^	13.44 ± 2.88 ^a^	0.35 ± 0.32 ^a^	17.70 ± 1.75 ^a^
PEF_1.5	48.95 ± 7.32 ^a^	−33.74 ± 17.25 ^a^	0.56 ± 0.13 ^ab^	0.54 ± 0.13 ^a^	27.35 ± 11.17 ^a^	16.88 ± 13.58 ^a^	0.35 ± 0.06 ^a^	18.84 ± 2.13 ^a^
PEF_2.0	45.23 ± 7.98 ^a^	−26.31 ± 5.64 ^a^	0.51 ± 0.05 ^b^	0.51 ± 0.03 ^a^	23.28 ± 5.25 ^a^	12.08 ± 3.41 ^a^	0.36 ± 0.03 ^a^	20.00 ± 3.30 ^a^

All values are expressed as the mean ± standard deviation (n = 14). Different letters (a, b) indicate significant differences in the same column (*p* < 0.05).

## Data Availability

The original contributions presented in this study are included in the article/Supplementary Materials. Further inquiries can be directed to the corresponding author.

## Supplementary Materials

The following supporting information can be downloaded at: https://www.mdpi.com/article/10.3390/foods14020148/s1, Video S1: Supplementary_Video_Shrimp_Peeling process.mp4.
